# Thickness Gradient in Polymer Coating by Reactive Layer-by-Layer Assembly on Solid Substrate

**DOI:** 10.1021/acsomega.3c05445

**Published:** 2023-09-26

**Authors:** Sezer Özenler, Ali Ata Alkan, Ufuk Saim Gunay, Ozgün Daglar, Hakan Durmaz, Umit Hakan Yildiz

**Affiliations:** † Department of Chemistry, Izmir Institute of Technology, Urla 35430, Izmir, Turkey; ‡ Leibniz-Institut für Polymerforschung Dresden e.V., Hohe Strasse 6, 01069 Dresden, Germany; § Department of Chemistry, Istanbul Technical University, Maslak 34469, Istanbul, Turkey; ∥ Department of Polymer Science and Engineering, Izmir Institute of Technology, Urla 35430, Izmir, Turkey

## Abstract

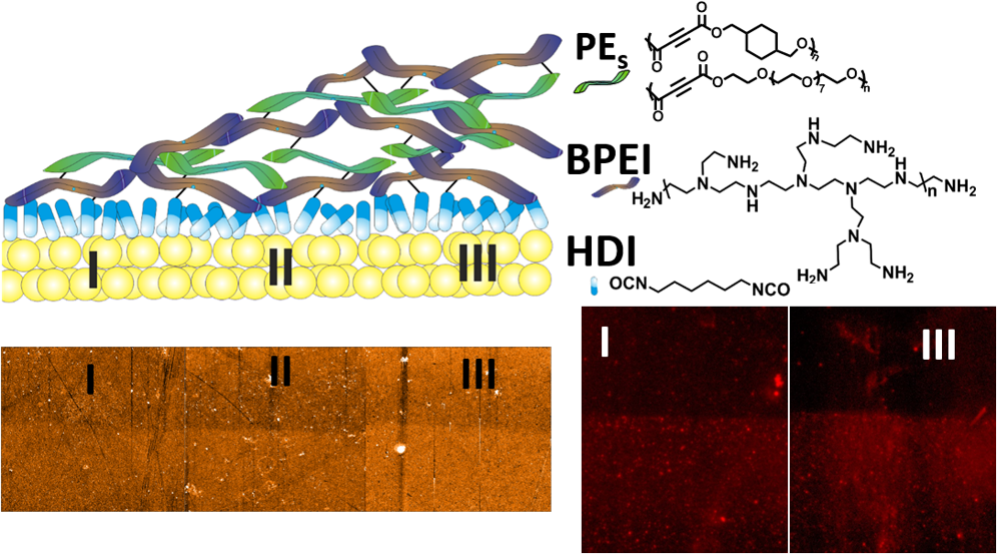

The study describes a simple yet robust methodology for forming gradients in polymer coatings with nanometer-thickness precision. The thickness gradients of 0–20 nm in the coating are obtained by a reactive layer-by-layer assembly of polyester and polyethylenimine on gold substrates. Three parameters are important in forming thickness gradients: (i) the incubation time, (ii) the incubation concentration of the polymer solutions, and (iii) the tilt angle of the gold substrate during the dipping process. After examining these parameters, the characterization of the anisotropic surface obtained under the best conditions is presented in the manuscript. The thickness profile and nanomechanical characterization of the polymer gradients are characterized by atomic force microscopy. The roughness analysis has demonstrated that the coating exhibited decreasing roughness with increasing thickness. On the other hand, Young’s moduli of the thin and thick coatings are 0.50 and 1.4 MPa, respectively, which assured an increase in mechanical stability with increasing coating thickness. Angle-dependent infrared spectroscopy reveals that the C–O–C ester groups of the polyesters exhibit a perpendicular orientation to the surface, while the C≡C groups are parallel to the surface. The surface properties of the polymer gradients are explored by fluorescence microscopy, proving that the dye’s fluorescence intensity increases as the coating thickness increases. The significant benefit of the suggested methodology is that it promises thickness control of gradients in the coating as a consequence of the fast reaction kinetics between layers and the reaction time.

## 1 Introduction

In interface engineering, precise thickness control and the fabrication of nanostructured surfaces are of particular importance because such manipulations are required for smart materials, self-cleaning surfaces, and antifouling surfaces, among other things.^1,2^ A variety of coating modalities are available for various applications by using manufacturing procedures, including spin coating, flow coating, and mask-assisted UV curing. The surface modification potential of reactive layer-by-layer synthesis (rLBL) appears to be quite promising as well. Although both LBL and rLBL involve numerous processing steps, the simplicity of both approaches is appealing to researchers. Electrostatic interactions are used in the LBL process, and gradient surfaces have anisotropic qualities because their chemical compositions change gradually over time, resulting in a variety of physical and biological characteristics on a single surface.^3,4^ Using deposition by the plasma copolymerization approach, Coad et al. produced several gradient polymers that might be used for research into surface topology and stiffness variations.^5,6^ A remarkably uncomplicated technique was reported by Li et al., which included placing the substrate at a tilting angle along the [CuIIL]/[CuIL] boundary for the diffusion control of electrochemically mediated atom transfer radical polymerization (eATRP) for surface modification using a gradient poly(3-sulfopropyl methacrylate potassium) brush.^7^ Using surface-initiated activators that were regenerated by electron transfer in ATRP, Tu et al. recently created a poly(polyethylene glycol methacrylate) (PEGMA) gradient surface using poly(ethylene glycol) methyl ether methacrylate. Following postmodification with Cys-Arg-Gly-Asp peptides, the gradient surface was scanned for cell density, which revealed a progressive drop in cell adherence.^8^ Additionally, novel and outstanding approaches have recently been developed, such as bottlebrushes and capillary microfluidic-assisted gradient polymer brushes.^9,10^ However, although high-throughput gradient surfaces are of significant interest in tissue engineering, biosensors, and particle sorting using diverse approaches, the production of gradient LBL films has been reported in only a few publications.^11,12^ As part of their investigation into multilayer formation by LBL deposition of a cationic and an anionic polymer on a gradient polymer brush, Shida et al. combined electrochemistry with ATRP to create a new technique called electrochemical atom transfer radical polymerization. The multilayer LBL film has a gradient height profile, as revealed by the thickness measurement of the film.^13^ Further, a gradient electrostatic LBL made of hyaluronan (HA) and poly(l-lysine) (PLL) was created by means of a microfluidic device to replicate an anisotropic cell microenvironment, which consists of distinct physical and biochemical areas. According to the findings, the changing physical (stiffness) and biochemical signals in gradient LBL can regulate cell spreading along the length of the gradient.^14,15^ Moreover, Sailer and Barrett created 2D gradient films covering all pH and salt combinations for both poly(allylamine hydrochloride) and poly(acrylic acid), allowing them to optimize high-throughput cellular screening on a single 7 cm square silicon wafer, which previously required 10 000 individual film samples.^16^ Compared with gradient electrostatic LBL, the rLBL approach offers four major benefits, namely, (i) the covalent bond offers excellent stability, (ii) rLBL does not require additional cross-linking, (iii) rLBL may be used in aqueous or organic reaction types, and (iv) the latest deposited layer does not disassemble the previously deposited layer.^17,18^ Using rLBL assembly with the aza-Michael reaction of branched polyethylenimine (BPEI) and polyester (PE) on the isocyanate-functionalized gold surface, our recent work showed that a 400 nm robust electroactive nanogel coating structure, allowing effective permeability mass and charge transfer, can be created. Results from X-ray photoelectron spectroscopy (XPS) depth profiling revealed that both polymers were present in each layer due to the presence of multiple functional groups in addition to the BPEI and PE intense layers of the nanogel.^19^ When interacting with surfaces, nanosized objects do not “feel” the presence of a gradient in the same way that larger objects do. As a result, high gradients at the nanometer scale are also required.^20−22^ In addition, one of the most important features of gradient assembly is obtaining regions with very different properties on a single substrate and readily finding the optimum properties of the surface for the desired application. In this work, we demonstrate how to construct a gradient film using rLBL assembly, which allows the integration of pre- and postfunctionality for the first time, thanks to PE structures. PEs with an electron-deficient triple bond, PE_CH_ and PE_PEG_, which were synthesized from the diols 1,4-cyclohexanedimethanol and octa-ethylene glycol, respectively, were grafted by nanogradient rLBL assembly on the isocyanate-functionalized gold surface using the BPEI method. Because BPEI and PE contain many functional groups on the gold surface, nanogradient rLBL may be used to regulate the behavior of nanometer-scale continuous gradient macromolecules on the surface. The approach produces a covalently linked homogeneous and durable gradient structure that can be easily adapted and repeated on a gold surface in a straightforward manner. Simple dip-coating methods provide high-precision thickness control over a vast surface area, which is a significant benefit of the suggested methodology.

## 2 Result and Discussion

**Scheme 1:**
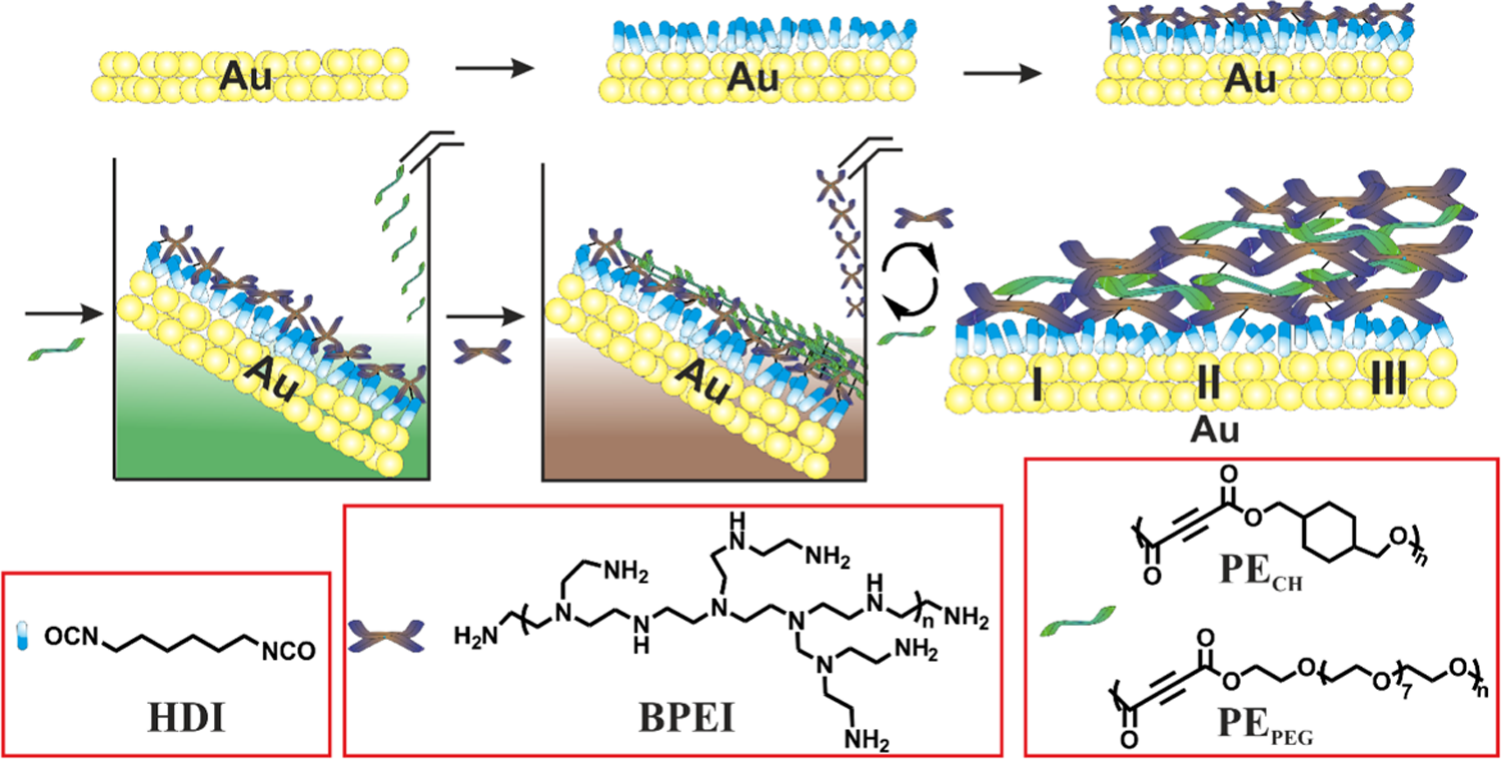
Fabrication of Nanogradient rLBL Assembly on a Gold Surface

Scheme 1 provides a schematic representation of nanogradient rLBL assembly based on the rLBL assembly method on a gold surface, the basis of which was detailed in a recent study.^23^ Initially, BPEI is grafted onto a hexamethylene diisocyanate (HDI)-modified gold surface via urea bond formation between the isocyanate and NH_2_ groups; however, numerous NH/NH_2_ functional groups remain unreacted on the surface. Next, the substrate is positioned at an angle of 45° to the plane, and PE in chloroform is added dropwise (0.5 mL/min) at room temperature and in an ambient atmosphere. Simultaneously, the alkyne bonds of PE give an aza-Michael reaction with the NH/NH_2_ groups on the surface. After the washing step, BPEI is added dropwise to the gold surface. The gradient rLBL film is formed after the sequential dropwise incubation step, and nanometer-scale macromolecule regions (Au, I, II, and III) are formed on the surface with various roughness and Young’s modulus values (Figure S1). The main reason for the formation of regions with different properties is that the gold surface is positioned at an angle of 45° to the plane; therefore, the surface is exposed to the reactive polymers (PE and BPEI) for different durations. The accumulation is higher on the surface that is exposed to the reactive polymer for longer, while the assembly is lower with less exposure time. Moreover, the gradient structure could also be formed because PE and BPEI, which have numerous functional groups, establish a continuous network-like structure on the surface.

**Figure 1:**
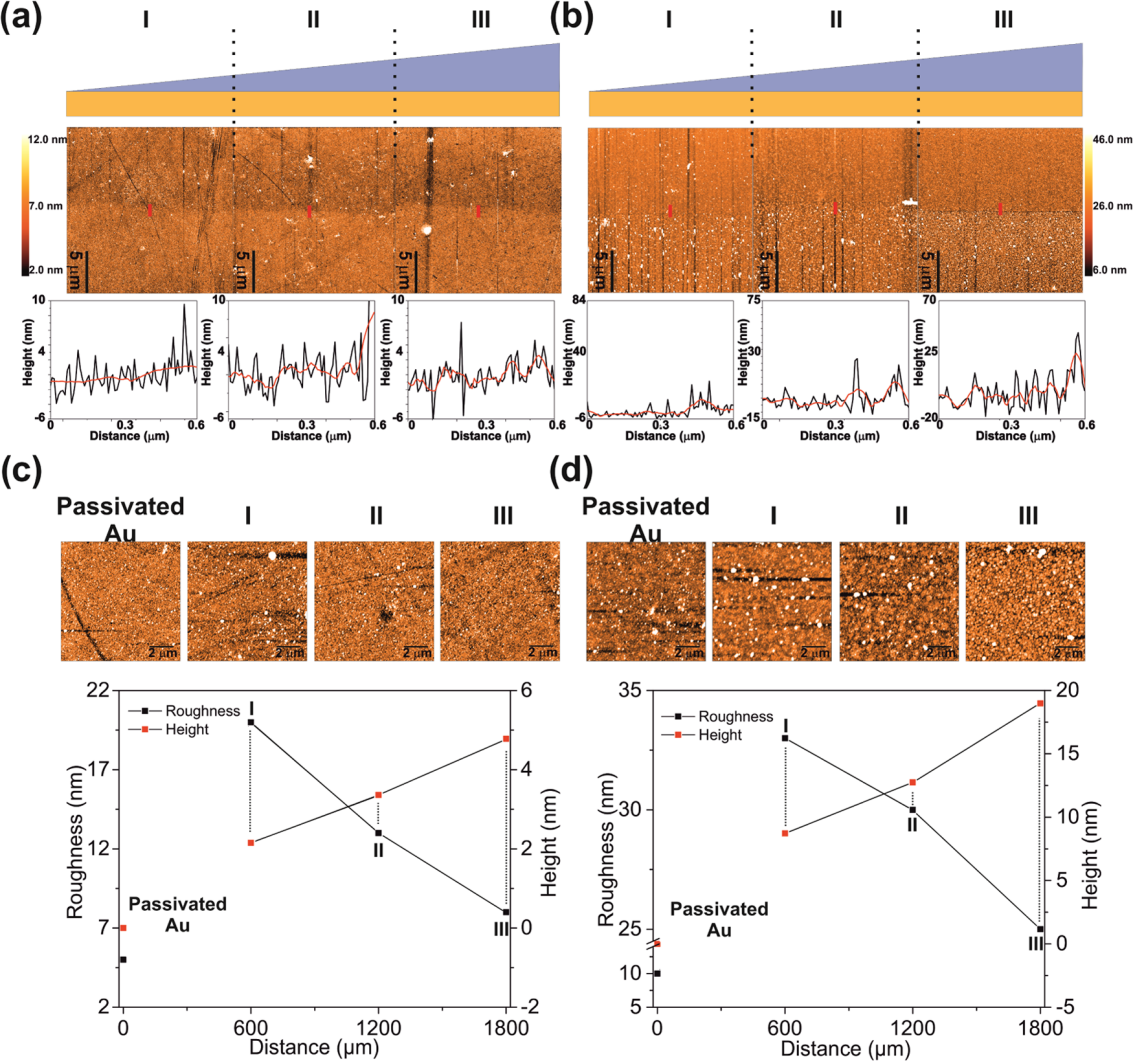
Schematic representation of regions I, II, and III (from thinner to thicker). AFM topography images (the images on the left were passivated with 11-mercapto-1-undecanol) and cross sections (short red lines) of regions I, II, and III of the (a) PE_CH_/BPEI binary system and (b) the PE_PEG_/BPEI binary system. Magnified AFM topography images of the Au region and regions I, II, and III of (c) the PE_CH_/BPEI binary system and (d) the PE_PEG_/BPEI binary system of height-roughness versus distance.

Figure 1a,b shows the AFM topography images of regions I, II, and III of the PE_CH_/BPEI and PE_PEG_/BPEI binary systems. These regions were measured on the same polymer line at a distance of almost 600 μm (Figure S2). In addition, the gold surface was passivated by a vertical rod-shaped patterned PDMS using 11-mercapto-1-undecanol to determine the polymer thickness.^19,23^ Unlike general gradient studies, this study used AFM instead of ellipsometry to determine the polymer thickness. The polymer height increased smoothly from regions I to III (Figure 1a), while an increase was observed in the spherical polymer structures in Figure 1b (see also Figure S3). The reaction on the surface is based on aza-Michael addition the most critical parameter. In addition, we concluded that concentration is an affecting parameter to control the yield; however, tilt angle was also a critical parameter in gradient formation (see Table S3). Regions I, II, and III of PE_CH_/BPEI have average height variations of 2.1, 3.3, and 4.7 nm, respectively. The PE_PEG_/BPEI binary system has height differences of 8.7, 12, and 18 nm in regions I, II, and III regions, respectively. In addition, the slope of PE_CH_/BPEI was 0.0022 nm/μm, as obtained from a linear fit (*R*^2^ = 0.99), while the slope of PE_PEG_/BPEI was 0.0086 nm/μm (*R*^2^ = 0.98). In Figure S4, the black dashed line (the lowest points of the cross sections) is used as a reference for height comparison, which is the intersection of passivated and nanogradient LBL. The AFM results confirm that nanogradient rLBL was obtained with nanometer-scale continuous macromolecules on the gold surface. Furthermore, Figure 1c,d shows a combined plot of the average height and roughness variation of the passivated Au region and regions I, II, and III, together with the corresponding magnified AFM topography images on top of the graphs. Figure 1c shows that the roughness decreases as the polymer thickness increases. The roughness variation was found to be similar to that obtained in a previous study, showing that an increase in gold nanoparticles (GNPs) caused a roughness decrease because of the gradual increase in GNP density with gradient LBL deposition.^13^ Like the PE_CH_/BPEI system, the PE_PEG_/BPEI binary system indicated that the roughness of the nanogradient rLBL assembly decreases as the thickness increases (Figure 1d). As a result, PE_PEG_/BPEI was slightly rougher than PE_CH_/BPEI because the former has more spherical polymer structures on its gold surface.

**Figure 2:**
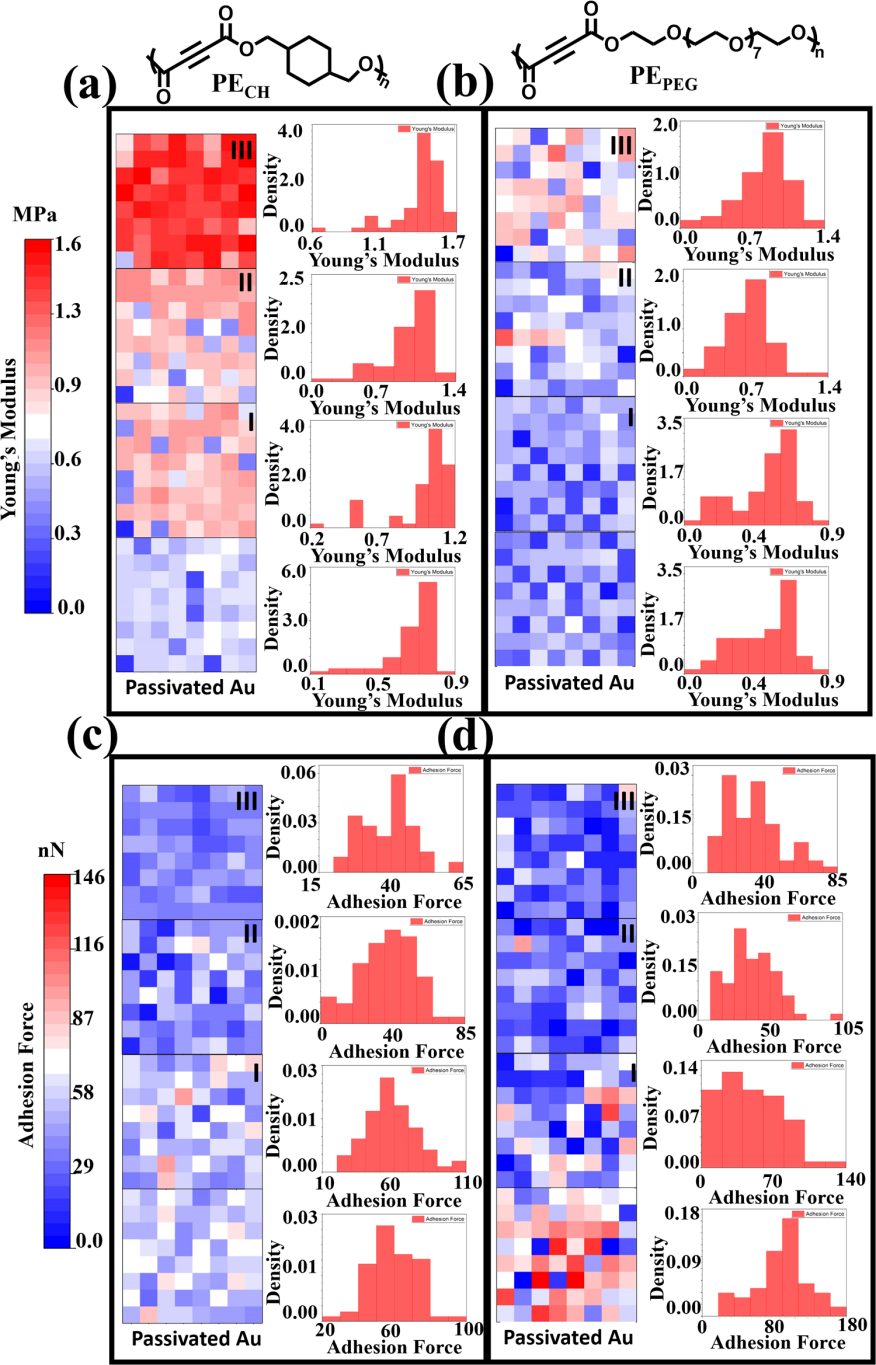
(a, b) Young’s modulus map of the passivated Au region and regions I, II, and III of PE_CH_/BPEI (left) and PE_PEG_/BPEI with the corresponding histogram (right). (c, d) Adhesion force map of the passivated Au region (left) and regions I, II, and III of PE_CH_/BPEI and PE_PEG_/BPEI with the corresponding histogram (right).

Nanomechanical characterization experiments were used to confirm the generation of the Young’s modulus and the adhesion force gradient. Maps of Young’s modulus and the adhesion force, together with the corresponding histogram of the passivated Au region and regions I, II, and III of PE_CH_/BPEI and PE_PEG_/BPEI, are shown in Figure 2. The transition from blue to red on the maps represents increasing values of the Young’s modulus and the adhesion force. The proposed methodology can be considered a fabrication method for understanding the effect of molecular-level interactions of nanometer-thick films on mechanical behavior. Regions I, II, and III of PE_CH_/BPEI have an average Young’s modulus of 1.0 ± 0.2, 0.9 ± 0.2, and 1.4 ± 0.2 MPa (passivated Au 0.6 ± 0.2), respectively. The PE_PEG_/BPEI binary system has Young’s modulus of 0.5 ± 0.2, 0.6 ± 0.2, and 0.8 ± 0.2 MPa (passivated Au 0.4 ± 0.2) in regions I, II, and III, respectively. Young’s modulus of the thin and thick regions is varied from 0.50 to 1.4 MPa, which increases mechanical stability with the increasing coating thickness. As a result, coating thickness is directly correlated to the improvement of Young’s modulus, which affects the nanomechanical properties significantly. According to the histograms, the Young’s modulus values of PE_CH_/BPEI and PE_PEG_/BPEI increase with an increasing thickness gradient (with a slope of, respectively, +0.0004 and +0.003 MPa/μm). The reason for these similar trends may be that larger amounts of reactive polymers are covalently bonded with an increase in polymer accumulation so that the polymers can form a more rigid structure. In addition, the effect of the stiffness of polyelectrolyte multilayer films on cell adhesion was investigated by varying the concentration of the 1-ethyl-3-(3-(dimethylamino)propyl) carbodiimide (EDC) cross-linker.^24^ While the film stiffness increased in the range of 200–600 kPa as a result of an increasing EDC concentration, MC3T3-E1 preosteoblastic cells attached and spread better in areas with high stiffness.^14^ Furthermore, the Young’s modulus value of PE_PEG_/BPEI is slightly lower than that of the PE_CH_/BPEI value. The reason for the low Young’s modulus value in the PE_PEG_/BPEI binary system could be that the ethoxy structure provides the polymer backbone with more flexibility. Using AFM, Huang et al. investigated the nanomechanical properties of PEG monolayers (<20 nm) on SnO_2_ nanofibers in liquid. The results indicated that the mechanical properties could be tuned from 5 MPa to 700 kPa by altering the molecular weight of the PEG films. The PEG films with a higher molecular weight showed a smaller Young’s modulus due to a higher expansion ratio.^25^ On the other hand, according to the histograms, the adhesion force values of PE_CH_/BPEI and PE_PEG_/BPEI decrease with an increasing thickness gradient (with a slope of, respectively, −0.0159 and −0.14 nN/μm). These similar trends might be attributed to the accumulating covalently bonded polymers creating more stable macromolecule regions. The mean values of Young’s modulus, the applied force, and the adhesion force of the Au region and regions I, II, and III of PE_CH_/BPEI and PE_PEG_/BPEI are given in Tables S1 and S2. In addition, the force–distance graphs representing the mean value of each region are presented in Figure S5. The results of Young’s modulus and the adhesion force prove that the gradient is fabricated with nanometer-thickness precision. In conclusion, as a result of the reaction between the increasing amount of PE (PE_CH_ and PE_PEG_) and BPEI, a gradient in Young’s modulus and the adhesion force was obtained without any additional cross-linking agents.

**Figure 3:**
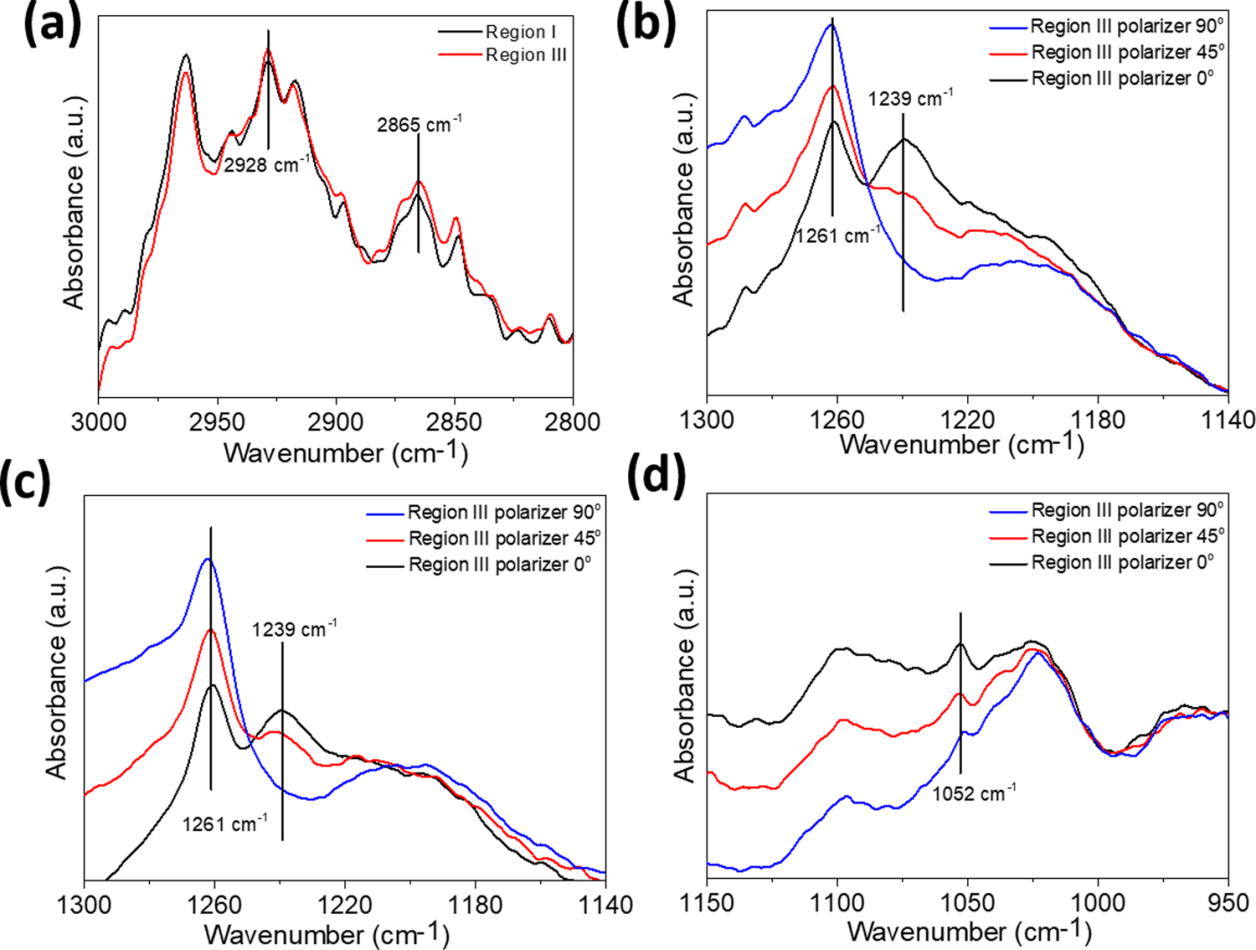
(a) FT-IR spectrum of regions I and III of the PE_CH_/BPEI binary system. (b) FT-IR spectrum of region III of the PE_CH_/BPEI binary system, with *p*-polarizer settings of 0, 45, and 90°. (c, d) FT-IR spectra of region III of the PE_PEG_/BPEI binary system, with p-polarizer settings of 0, 45, and 90°.

The extent and orientation of the binding of PEs were observed by FT-IR measurements of regions I, II, and III. Almodóvar et al. demonstrated that a percentage decrease in the COO^–^ band proves a decrease in cross-links with the gradient concentration of 1-ethyl-3-(3-(dimethylamino)propyl) carbodiimide (EDC).^14^ Additionally, Lee et al. investigated the hydroxyl group of poly(2-hydroxyethyl methacrylate) (PHEMA) at 3350 cm^–1^ to indicate that the density of PHEMA gradually decreases as the distance increases.^25^ Similarly, reflection–absorption infrared and Raman spectroscopy were used to determine the gradient difference.^26,27^ The FT-IR spectrum of the nanogradient rLBL assembly of PE_CH_/BPEI on the gold surface is presented in Figure 3a,b. The peaks in Figure 3a at 2928 and 2865 cm^–1^ could be attributed to symmetric and asymmetric cyclohexyl groups on the PE_CH_ backbone. At 2928 cm^–1^ the intensity variation in region III is 2% higher than that in region I, while at 2865 cm^–1^, the intensity variation in region III is 1.5% higher than that in region I. These slight changes in intensity are consistent with the nominal height difference between regions I and III in the AFM results (Figure 1c,d). In addition, when the polarizer is adjusted to 0°, the grid lines are parallel to the width of the polarizer mount and the transmitted IR radiation is perpendicular to this. Likewise, when the polarizer is adjusted to 90°, the grid lines are parallel to the length of the polarizer mount, and the transmitted IR radiation will be perpendicular to this in the VeeMAX III accessory. The FT-IR spectra of region III are shown in Figure 3b at polarizer settings of 0, 45, and 90°. When the polarizer changes from 90 to 0°, a new peak appears at 1239 cm^–1^, while the intensity of the peak at 1261 cm^–1^ decreases. The C–O–C asymmetric stretching vibration of the ester stretching of PE_CH_ and the amine C–N stretching of BPEI could be attributed to 1261 and 1239 cm^–1^, respectively. As a result of the 0° polarizer spectrum, it is determined that the BPEI is positioned horizontally on the gold surface. Moreover, it is found that the C–O–C ester groups of the polyesters exhibit perpendicular orientation to the surface, while the C≡C groups are parallel to the surface. A similar trend can also be seen in region III of the PE_PEG_/BPEI binary system in Figure 3c (see also Figure S6). In addition, the peak at 1052 cm^–1^, which can be attributed to the ether bond of the PE_PEG_/BPEI binary system, yields the most distinctive peak when the polarizer is at 0°. The orientation of the PE on the surface gives the highest value of the peak intensity at 2164 cm^–1^, which represents the carbon triple bond when the polarizer is at 0° (Figure S7).

**Figure 4:**
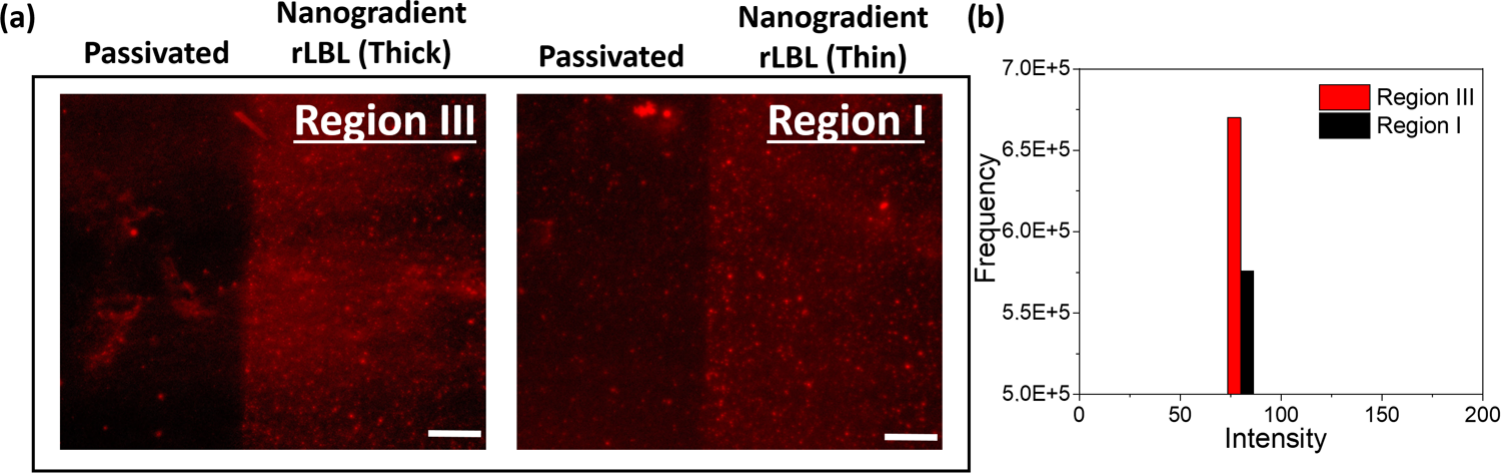
(a) Fluorescence image of regions III and I of the PE_PEG_/BPEI binary system after incubation with NH_2_-conjugated dye (scale bar 50 μm). (b) Frequency versus red-intensity graph.

Fluorescent images of the nanogradient rLBL film after 1 min incubation with NH_2_-conjugated dye show various fluorescence intensities depending on regions. Li et al. demonstrated that the fluorescence intensity of bovine serum albumin labeled with fluorescein isothiocyanate increases gradually with the *x*-direction by postmodification of the poly(acrylic acid) gradient on the poly(*N*-isopropylacrylamide) surface.^28^ In addition, Morgenthaler et al. showed that the intensity of fluorescently labeled streptavidin decreases along the gradient of biotinylated poly-l-lysine grafted with polyethylene glycol.^29^Figure 4 shows the fluorescent images of the nanogradient rLBL film after 1 min incubation with NH_2_-conjugated dye to understand the postmodification efficiency. A PE with electron-deficient triple bonds allows aza-Michael addition reactions with very high efficiency after 2 min without any catalysts. The reason for the difference in thickness between regions I and III is the amount of reacted PEs/BPEI on the gold surface. The thicker region III has more unreacted triple bonds, as it contains more PEs. After NH_2_-conjugated dye incubation, the difference in fluorescence intensity between regions I and III is consistent with the nanogradient rLBL film thicknesses. As shown in Figure 4b, the total fluorescence intensity difference between regions I and III is nearly 15%. This result demonstrates that the postmodification of the nanogradient rLBL film was accomplished for both regions. In conclusion, the nanogradient rLBL film can be postmodified with different amounts of NH_2_ groups between regions I and III on the gold surface.

## 3 Conclusions

This study described polymer gradient formation on flat substrates by rLBL assembly via the aza-Michael reaction between BPEI and an electron-deficient alkyne containing PE_PEG_ and PE_CH_. The gradient thickness was controlled by concentrations of stock polymer solutions and incubation time. The gradient profile was analyzed via AFM, revealing that a gradient from 0 to 20 nm was obtained in the coating along a length of 1800 μm. The nanomechanical characterization of the polymer coating showed that Young’s modulus of thin (region I) and thick regions (region III) varied between 0.50 and 1.4 MPa and that the adhesion force varied between 58 and 31 nN. Angle-dependent infrared characterization of the polymer gradient coatings confirmed that the C–O–C group was perpendicular to the plane, whereas the C–N and C≡C groups were parallel to the plane. The facile preparation of the polymer gradient was achieved by the rLBL strategy, which enables easy control of engineering surfaces for applications such as antifouling surfaces and biosensors.

## 4 Experimental Section/Methods

All chemicals were purchased from Sigma-Aldrich and used as received without further purification. Synthesis of PEs (PE_CH_ and PE_PEG_) was achieved via polymerization between acetylenedicarboxylic acid and diol compounds according to the published method.^30,31^ Nanosurf AFM was used for topographical (ElectriMulti75-G, static force) and force–distance (ElectriMulti75-G, force modulation -3 nm indentation) investigation of nanogradient rLBL assembly on gold surface (see Figures S9 and S10).^32^ Infrared measurements were carried out using VeeMAX III Variable Angle Specular Reflectance Accessory (3/8 in. mask) combined PerkinElmer Spectrum 100 FT-IR at 40° incident beam with setting 0, 45, and 90° Zn–Se p-polarizer. The NH_2_-dye procedure was based on incubating 1 min of NH_2_-conjugated dye on the gold surface. Following incubation, the gold surfaces were rinsed, and fluorescence imaging of the gold surfaces was conducted by utilizing Zeiss fluorescence microscopy with an exciting wavelength of 541 nm. The total intensity of the corresponding region of interest was calculated automatically by the ZEISS ZEN Microscopy Software (Zen 2 Core, see Figure S8).

General procedure for surface modification: The gold surface cleaning and passivation procedures were performed as described previously.^19^ The clean gold surface was incubated in 80 mM (35 μL) hexamethylene diisocyanate (HDI) solution in acetone (4.5 × 10^–2^ mm DBTDL as a catalyst) at 40 °C for 25 min. The gold surface was sonicated with plenty of chloroform to remove excess polymers. After HDI incubation, the gold substrate was incubated with 1.2 mM BPEI (40 mg, Mw: 25,000) solution in chloroform at 40 °C for 25 min. To obtain nanogradient rLBL assembly, the gold surface tilted 45° and the beaker charged 0.5 mL/min PE solution (18 mg, 3.4 mM) in chloroform at room temperature and ambient atmosphere. After the gold surface was completely immersed in the PE solution, the surface was sonicated with excess chloroform, and the same procedure was performed with the BPEI solution. The same incubation method was applied with PE and BPEI solutions once again (Table S3).

## Supporting Information Available

Nanogradient rLBL assembly scheme, microscope and AFM images of surfaces, Young’s modulus and adhesion force tables and force–distance graphs, FT-IR spectrum, and parameters of the experiments (PDF)
