# Supplementary material for: Thickness Gradient in Polymer Coating by Reactive Layer-by-Layer Assembly on Solid Substrate
Source: ACS Omega. 2023 Sep 26;8(40):37413–20. doi: 10.1021/acsomega.3c05445 (PMC10568690; doi:10.1021/acsomega.3c05445)
Supplement: Supplementary file 1 — ao3c05445_si_001.pdf [file ao3c05445_si_001.pdf]

Supporting Information

# Thickness Gradient in Polymer Coating by Reactive Layer-by-Layer Assembly on Solid Substrate

*Sezer Özenler[a,b*], Ali Ata Alkan[d], Ufuk Saim Gunay[c], Ozgün Daglar[c], Hakan Durmaz[c] and Umit Hakan Yildiz[a,d*]*

a Department of Chemistry, Izmir Institute of Technology, Urla, 35430 Izmir, Turkey

b Leibniz-Institut für Polymerforschung Dresden e.V., Hohe Strasse 6, 01069, Dresden, Germany

c Department of Chemistry, Istanbul Technical University, Maslak, Istanbul 34469 Turkey

d Department of Polymer Science and Engineering, Izmir Institute of Technology, Izmir 35430 Turkey

E-mail: hakanyildiz@iyte.edu.tr, oezenler@ipfdd.de

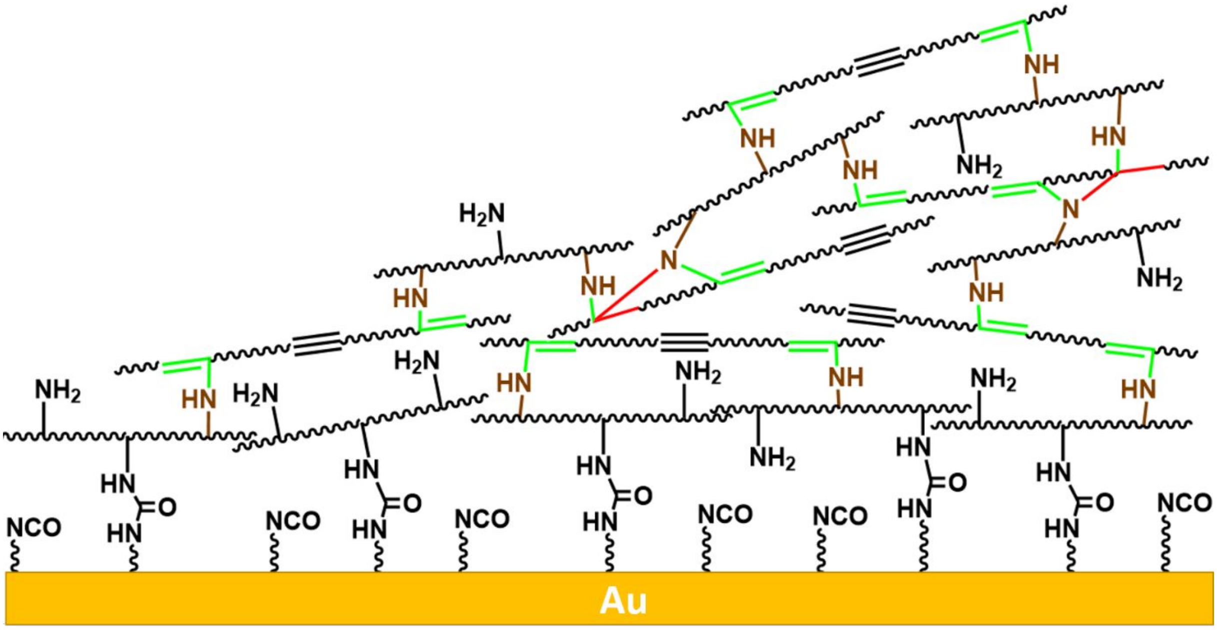


**Figure S1.** Fabrication of nanogradient rLBL assembly on a gold surface.

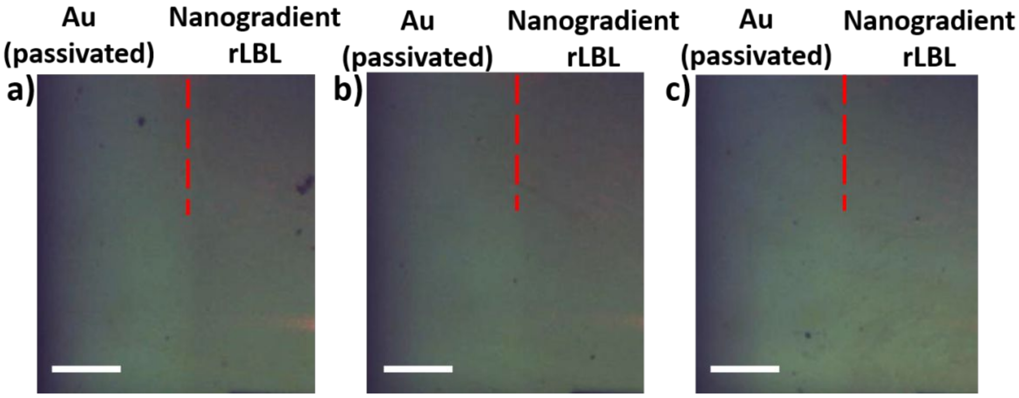


**Figure S2.** Microscope images of the $PE_{PEG}$/BPEI binary system: (a) region III, (b) region II, and (c) region I. Images are shown at 600 µm distances on the same polymer strip. (scale bar 100 µm).

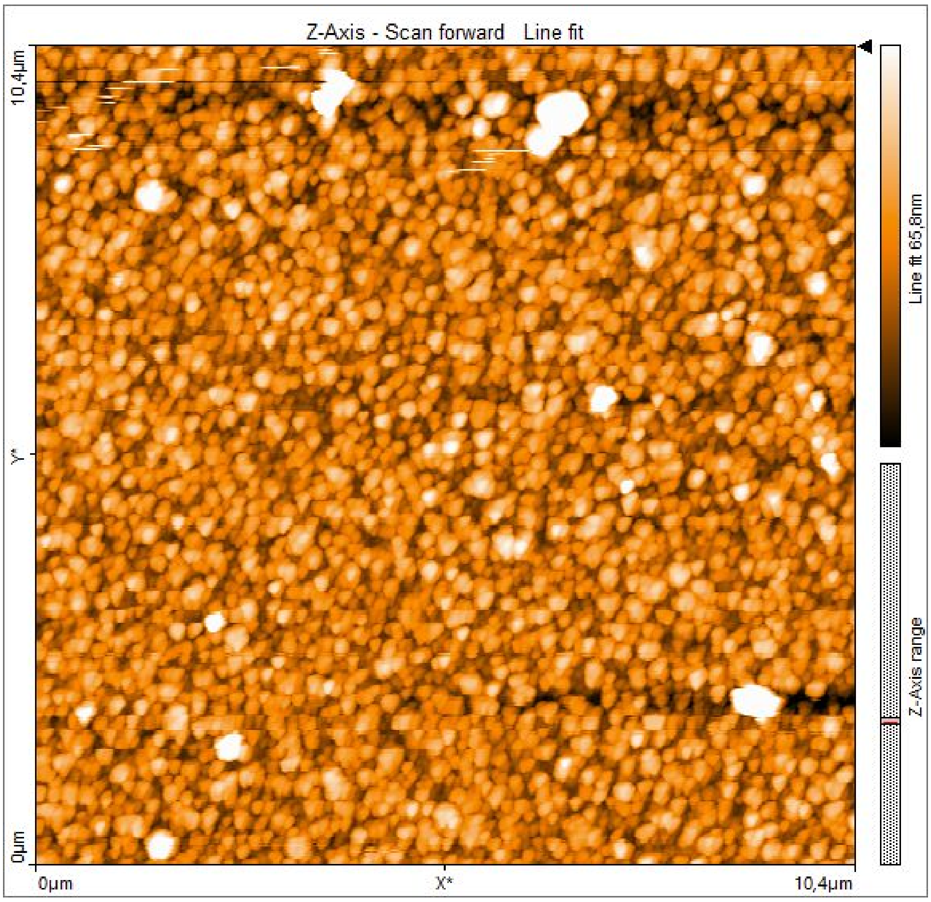


**Figure S3.** AFM images of region III of $PE_{PEG}$/BPEI.

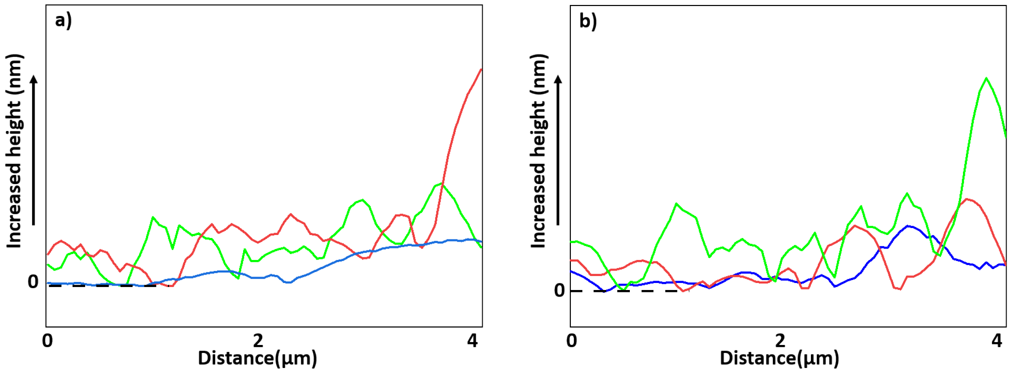


**Figure S4.** Overlap of cross-sections of three regions (a) $PE_{CH}$/BPEI binary system and (b) the $PE_{PEG}$/BPEI binary system. The blue is Region I, the red is Region II, and the green is Region III.

**Table S1.** Mean value of Young's modulus, applied force, and adhesion force of $PE_{CH}$/BPEI.

| | Passivated Au | Region I of $PE_{CH}$/BPEI | Region II of $PE_{CH}$/BPEI | Region III of $PE_{CH}$/BPEI |
|---|---|---|---|---|
| Young's modulus mean value (MPa) | 0.6 ± 0.2 | 1.0 ± 0.2 | 0.9 ± 0.2 | 1.4 ± 0.2 |
| Applied force mean value (nN) | 6.7E+1 ± 20 | 9.5E+1 ± 21 | 9.3E+1 ± 21 | 1.4E+2 ± 16 |
| Adhesion force mean value (nN) | 5.9E+1 ± 15 | 5.8E+1 ± 17 | 4.2E+1 ± 17 | 3.9E+1 ± 14 |

**Table S2.** Mean value of Young's modulus, applied force, and adhesion force of $PE_{PEG}$/BPEI.

| | Passivated Au | Region I of $PE_{PEG}$/BPEI | Region II of $PE_{PEG}$/BPEI | Region III of $PE_{PEG}$/BPEI |
|---|---|---|---|---|

| | | | | |
|---|---|---|---|---|
| Young's modulus mean value (MPa) | 0.4 ± 0.2 | 0.5 ± 0.2 | 0.6 ± 0.2 | 0.8 ± 0.2 |
| Applied force mean value (nN) | 5.0E+1 ± 20 | 5.2E+1 ± 18 | 6.5E+1 ± 21 | 8.2E+1 ± 20 |
| Adhesion force mean value (nN) | 7.8E+1 ± 18 | 4.8E+1 ± 29 | 3.5E+1 ± 19 | 3.1E+1 ± 19 |

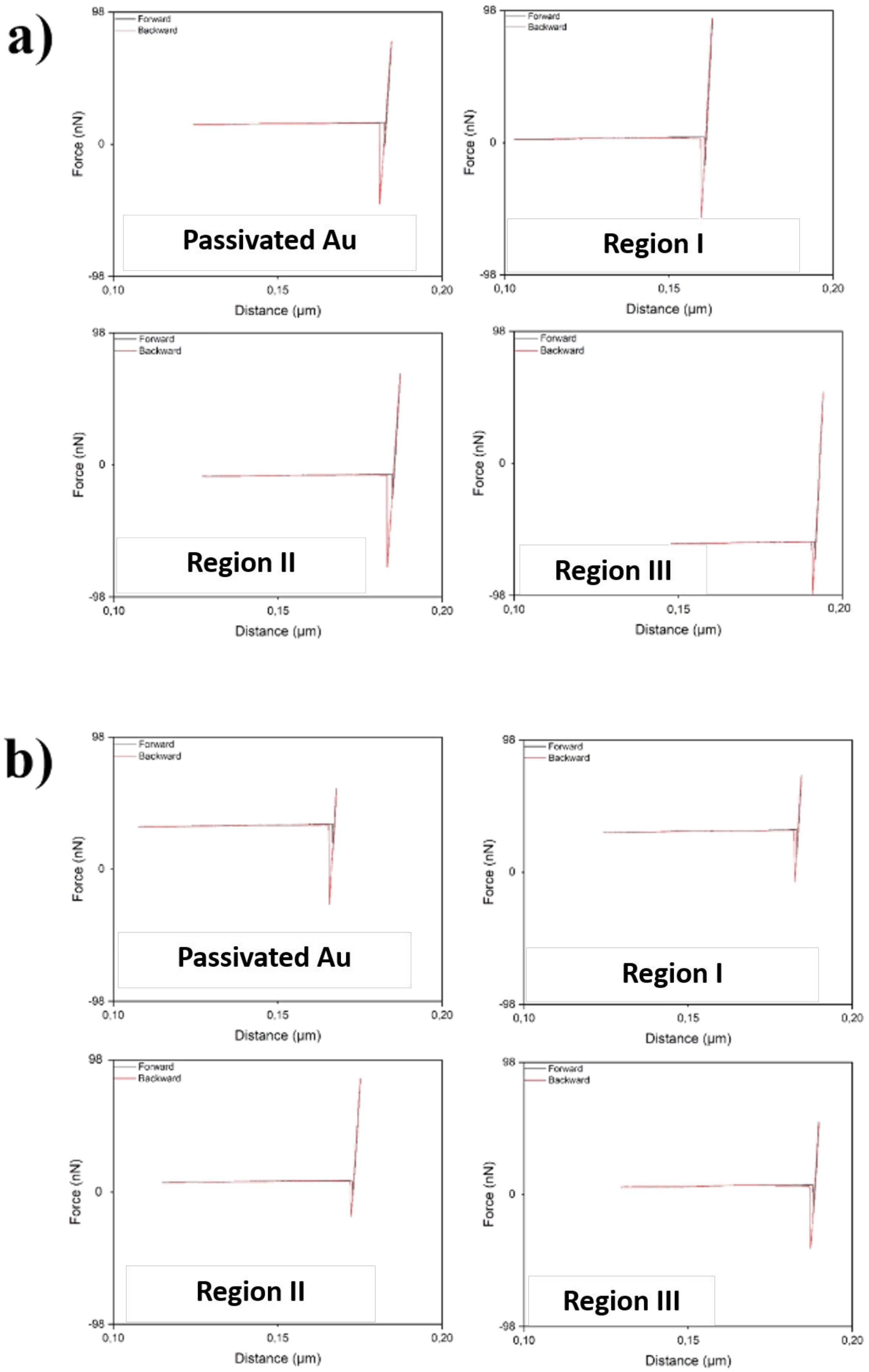


**Figure S5.** Force-distance graphs of (a) $PE_{CH}$/BPEI and (b) $PE_{PEG}$/BPEI.

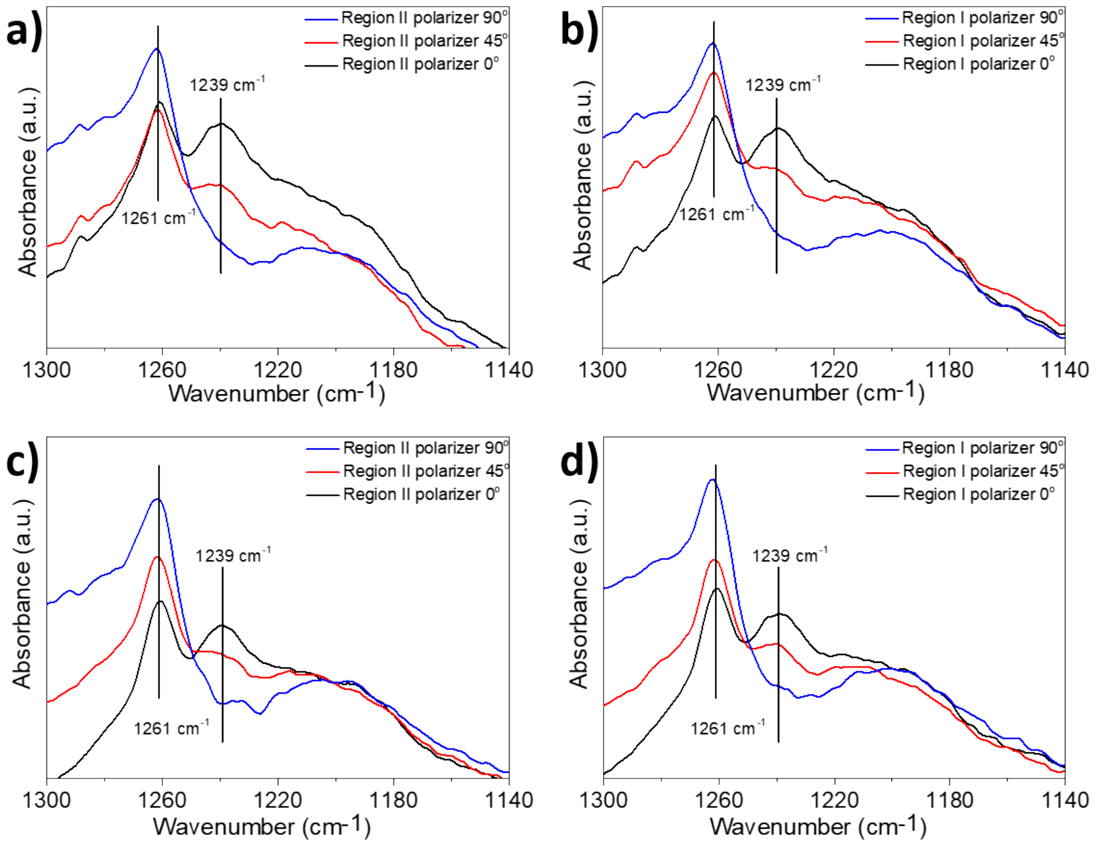


**Figure S6.** (a, b) FT-IR spectrum of regions II and I of the $PE_{CH}$/BPEI binary system, with p-polarizer settings of 0°, 45°, and 90°. (c, d) FT-IR spectrum of regions II and I of the $PE_{PEG}$/BPEI binary system, with p-polarizer settings of 0°, 45°, and 90°.

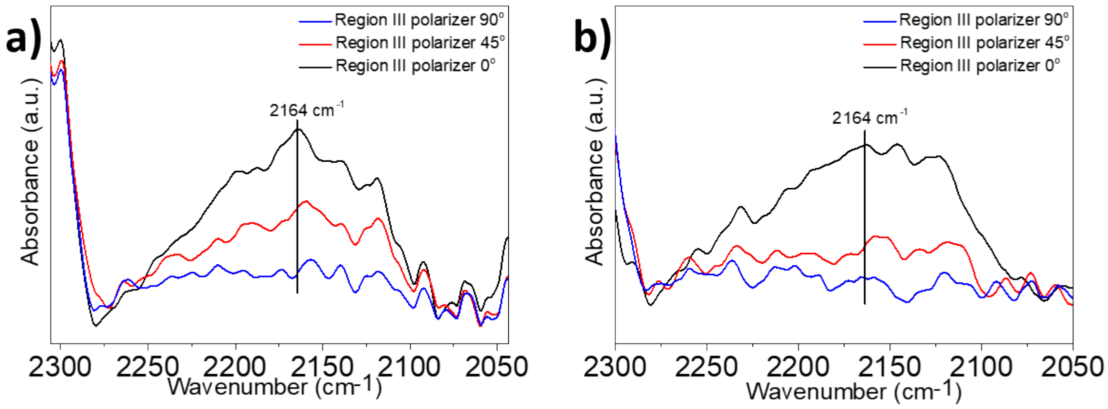


**Figure S7.** FT-IR spectrum of region III of (a) the $PE_{CH}$/BPEI and (b) the $PE_{PEG}$/BPEI binary system, with p-polarizer settings of 0°, 45°, and 90°.

**Table S3.** The parameters of the experiments.

| # | Branched Polyethyleneimine (BPEI) | Polyester (PE) | Tilt angle | Incubation time |
|---|---|---|---|---|
| 1 | 70 mg BPEI/5 ml $CHCl_3$ | 15 mg PE / 5 ml $CHCl_3$ | 90 degrees | 1.6 ml/minutes |
| 2 | 28 mg BPEI/5 ml $CHCl_3$ | 14 mg PE/5 ml $CHCl_3$ | 60 degrees | 1.2 ml/minutes |
| 3 | 20 mg BPEI/5 ml $CHCl_3$ | 10 mg PE/5 ml $CHCl_3$ | 60 degrees | 1.2 ml/minutes |
| 4 | 20 mg BPEI/5 ml $CHCl_3$ | 10 mg PE/5 ml $CHCl_3$ | 60 degrees | 2.4 ml/minutes |
| 5 | 20 mg BPEI/5 ml $CHCl_3$ | 10 mg PE/5 ml $CHCl_3$ | 60 degrees | 0.5 ml/minutes |
| 6 | 40 mg BPEI/5 ml $CHCl_3$ | 18 mg PE/5 ml $CHCl_3$ | 60 degrees | 1.2 ml/minutes |
| 7 | 40 mg BPEI/5 ml $CHCl_3$ | 18 mg PE/5 ml $CHCl_3$ | 45 degrees | 1.2 ml/minutes |
| 8* | 40 mg BPEI/5 ml $CHCl_3$ | 18 mg PE/5 ml $CHCl_3$ | 45 degrees | 0.5 ml/minutes |

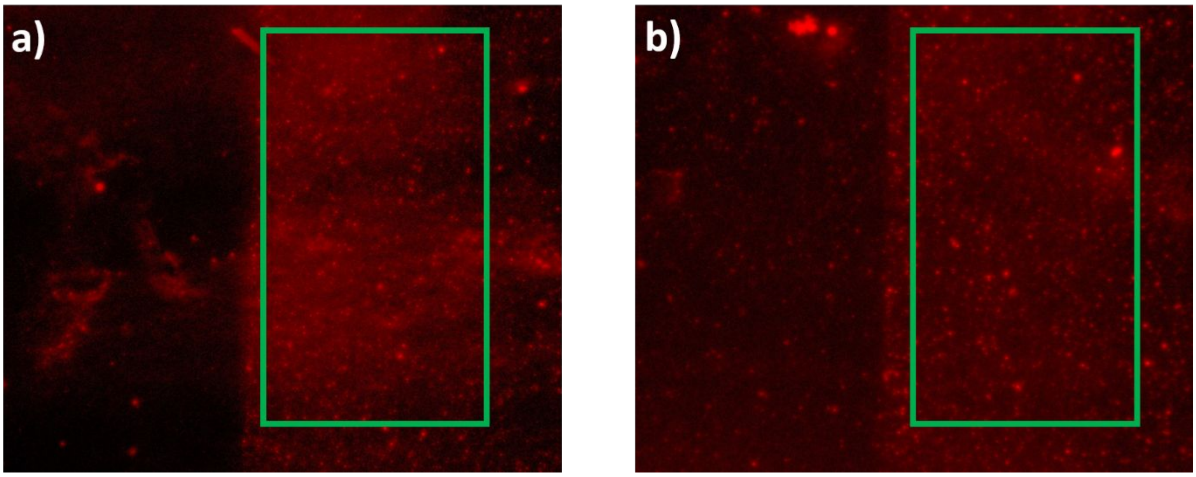


**Figure S8.** (a) The fluorescence image of regions III and (b) I of region of interest of the $PE_{PEG}$/BPEI binary system.

**Nanomechanical Characterization:**

Hetz Model;

$$F = \frac{4}{3}\frac{E}{1-\mu^2}\sqrt{R\delta_0^3}$$

Where,
F: Applied Force, which is taken from the spectroscopic technique via AFM Force Modulation.
E: Young's Modulus, which is used to characterize the material's elastic behavior.
$\mu$: Poisson's Ratio that corresponds the material anisotropic behavior.
$\delta$: Displacement of the tip between start of the deformation and end of the deformation. (This deformation is about to elastic deformation)
R: Radius of the tip

According to Hertz model, every raw data (Applied force and the displacement of the tip) is taken from the AFM software and evaluated by running custom made MatLab code. Raw data comes from AFM software just for one approached point for each region in the mapping and gives one applied force data for each region. After evaluation by using MatLab, there are no statistical approach for regions of map due to absence of the statistical sampling. For Young's Modulus maps (collection of different regions), young's modulus distribution and the mean values of the different surfaces are given to give an access for the statistical evaluation. Precision of the calculated values according to Hertz Model is originated due to taken data precision and the high resolution of the MatLab. All values are measured with highly resolved spectrometer CoreAFM) and calculated by mathematical models (MatLab custom made code).

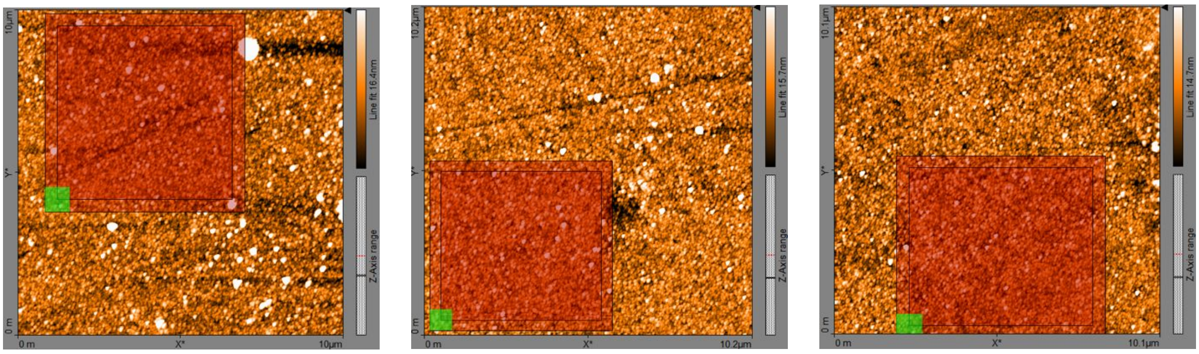

**Figure S9.** Selected Regions for F-d Penetration for $PE_{CH}$ (from region I to III)

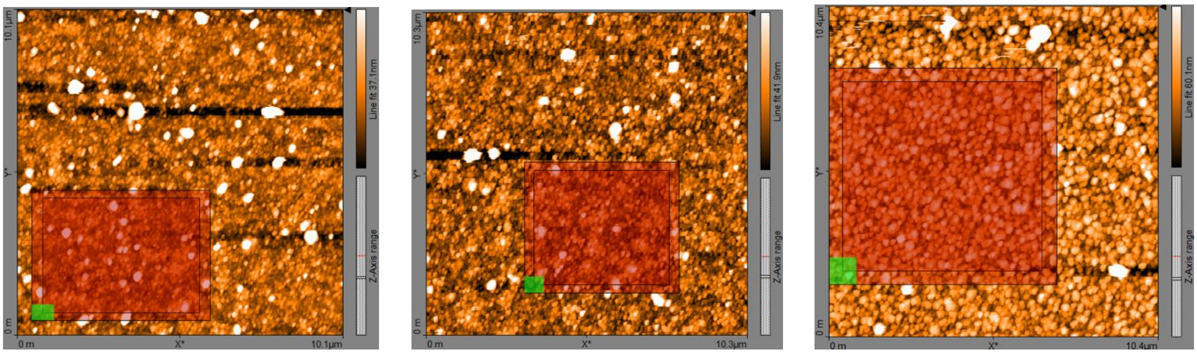

**Figure S10.** Selected Regions for F-d Penetration for $PE_{PEG}$ (from region I to III)

Selected areas in Figures S9 and S10 represent the AFM tip penetration regions. Each area is divided into 64 sub-regions to make statistical assurance about the spectroscopic method. 64 regions indicate 64 points where penetration is done. Penetration regions are ordered as shown above. Every point is subjected to a specified force and deformed by this applied force. When each penetration is done, an adhesion force occurs with the withdrawn process. These applied forces cause an elastic deformation and the linear relation between those applied forces and deformations gives Young's Modulus of the surface. To make statistical control, 64 points were subjected to probability analysis.
